# The influence of multi-layered varnishes on moisture protection and vibrational properties of violin wood

**DOI:** 10.1038/s41598-019-54991-5

**Published:** 2019-12-09

**Authors:** Sarah L. Lämmlein, David Mannes, Bart Van Damme, Francis W. M. R. Schwarze, Ingo Burgert

**Affiliations:** 1 Swiss Federal Laboratories for Materials Science and Technology (Empa), Dübendorf, Switzerland; 2 Swiss Federal Institute of Technology Zürich (ETH Zürich), Zurich, Switzerland; 3 Paul Scherrer Institute (PSI), Villigen, Switzerland; 4 Swiss Federal Laboratories for Materials Science and Technology (Empa), St. Gallen, Switzerland

**Keywords:** Engineering, Materials science

## Abstract

Violin varnishes are known to affect both moisture absorption and vibrational properties of violin wood. However, traditional multi-layered varnish systems suffer from substantial wear as a result of intensive use, which calls for deeper understanding of the specific impact of individual layers. Using sophisticated *in-situ* neutron imaging and vibrational modal analysis, we show how wood sorption and vibrational behavior of tonewood depend on the build-up of the varnish system. The results demonstrate the protective effect of complete coatings and emphasize that strongly worn regions cannot accomplish the function as an effective moisture barrier, which might pose a risk for frequently played or aged string instruments. Furthermore, the build-up of the varnish system affects the vibrational properties of the tonewood, influencing its final sound quality. This delicate interplay should be considered both for the handling of antique and aged violins and for the production of modern high-quality instruments.

## Introduction

Many luthiers apply specific and individual varnish procedures to influence the aesthetic appearance and occasionally the sound of a violin. Generally, a coating consists of multiple layers, which in turn comprise different materials. Considering the great variation of applied materials and varnishing methods, it is impossible to refer to a standard procedure. If a darkening of the wood surface is required, a stain is applied in the first step. The subsequent wood penetrating treatments are described as primer, impregnation, sealer, ground or filling, which are different terms to describe similar treatments and should restrain the following layers from penetrating into open wood cells^1–3^. Continuous layers applied on top of the pretreatments are commonly referred to as varnishes. However, varnish often also denotes the entire coating system as no consistent terminology has been used up to date^2–4^. To protect knowledge, luthiers in the past and present avoid to publicly disclose their varnishing procedures^2,5–7^ which has resulted in a loss of knowledge about the antique materials and methods used by the Cremonese masters^8^. Therefore, several chemical investigations of the varnish materials and methods^3,9–11^ and studies on the stratigraphy^2,5,11^ of antique instruments have been conducted.

While the influence of a specific coating system on the sound quality of a violin is not yet fully understood, its general influence on vibrational properties is commonly accepted, as the application of a varnish typically increases damping and radial stiffness and reduces longitudinal stiffness of wood strips^12–14^. Another clear function of a varnish is the protection of the violin against general wear and relative humidity influences. In contrast to the chemical and vibrational studies, the general protective behavior of violin vanishes against moisture changes has, however, not been a main research objective to-date. The varnish’s function as a moisture barrier is of particular importance as the wood moisture content (MC) influences the mechanical properties of wood and results in dimensional changes owing to moisture uptake and release^15–17^. The swelling and shrinkage of wood can result in board cupping and moisture gradients causing internal stresses, which may lead to crack formation and possible failure of the instrument^18^.

A former study has shown that little diffusion is taking place through the varnish material itself^19^. Further, the opened f-holes and unvarnished inner surfaces depict a general sorption capacity of the violin^20^. Accordingly, a varnished violin showed only half of the mass increase due to moisture uptake compared to an unvarnished violin^21^. Structural deformations due to moisture gradients were measured in terms of board cupping effects caused by the one-sided varnish application^22^. However, a study of seven different fillers showed that none of them acted as effective moisture barriers, where after one week all treated samples showed a similar wood moisture uptake when compared to an untreated reference sample^23^.

**Figure 1:**
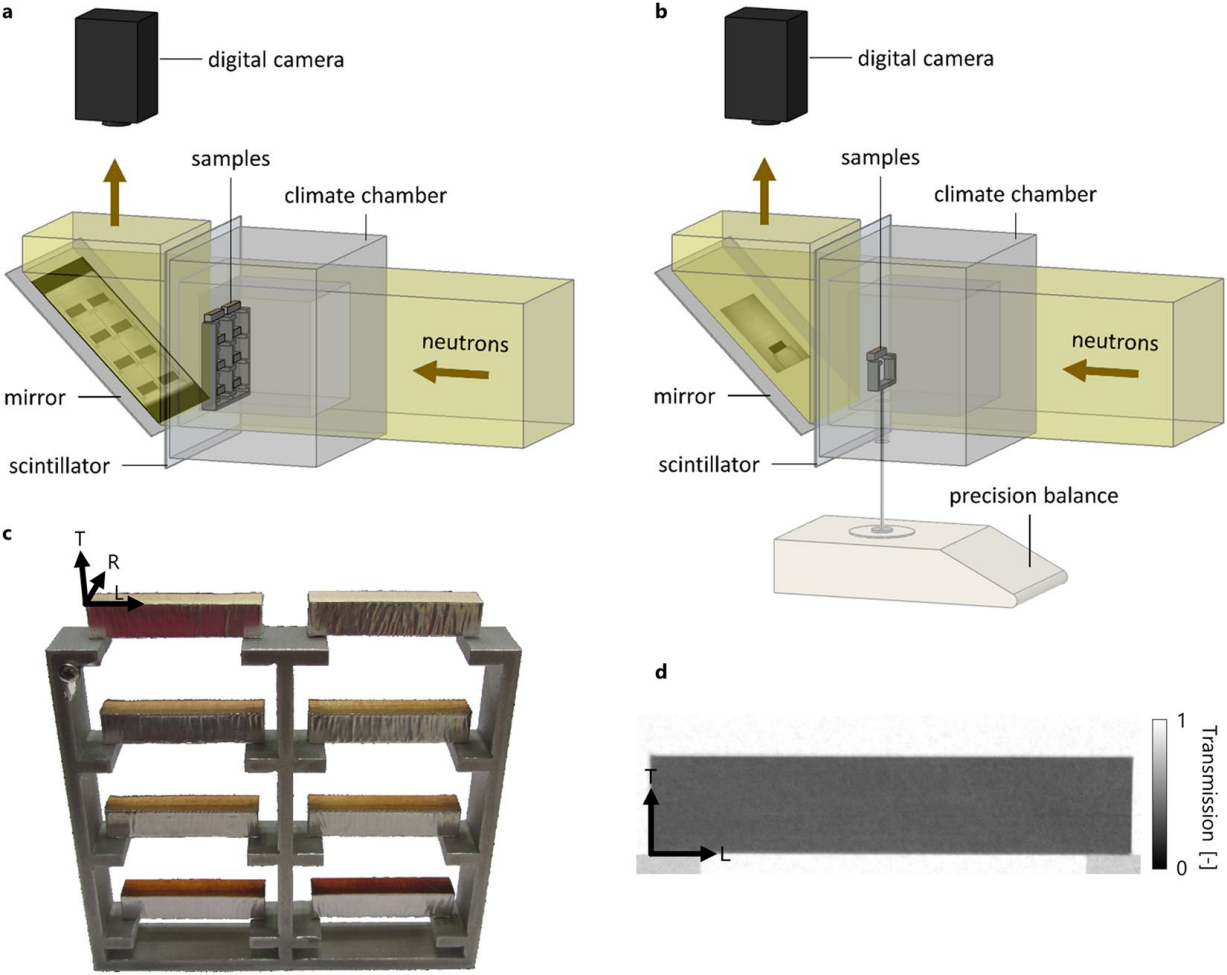
Schematic drawing of the neutron imaging setup used for (**a**) comparison of different varnish systems and (**b**) *in-situ* MC changes validation with precision balance weight measurements. (**c**) Varnished spruce wood samples on the frame illustrating the different investigated coating systems that were applied to the top surface (from top to bottom and left to right): (i) Vernice Bianca (B), (ii) unvarnished reference sample, (iii) sodium nitrite solution (N), (iv) N + sealing (S), (v) N + grounding (G), (vi) N + S + G, (vii) N + S + G + alcohol shellac varnish (A) and (vii) N + S + G + oil copal varnish (O). On basis of the intensity image of an individual sample, the corresponding and corrected (**d**) transmission image is gained, which allows the calculation of the spatial MC changes over time during the sorption (from 35% RH to 95% RH for 5 h) and desorption process (subsequently back to 35% RH for another 5 h).

These diverse and inconsistent results on a topic of immense cultural and historical relevance call for a more systematic study on the protective nature of different varnish systems and compositions. Frequently played and aged instruments show a wear pattern due to varnish degradation after extensive handling and playing by the violinist^2,11^. Body contact results in wear and exposes the surface of the violin to sweat and moisture^24,25^. Taking these alterations of the varnish system and their imitation on modern violins into account, the contribution of individual varnish treatments to the impact of the coating system is of particular interest. For this purpose, we have studied the influence of common traditional European varnish materials and coating build-ups on the sorption process of the underlying wood to understand how instruments react to changes in relative humidity. Neutron imaging is known as a convenient technique to investigate moisture content changes and liquid water transport in wood^26–29^ and its suitability to study the influence of coatings on the sorption dynamics has been demonstrated^30^. By using *in-situ* neutron imaging, we could non-destructively quantify and localize wood moisture gradient changes over time with a spatial resolution of several tens of micrometers (Fig. 1). For this purpose the impact of seven different coatings on the sorption process, ranging from simple pretreatments to complex varnish systems, was studied in comparison to unvarnished reference samples (Fig. 1c). As an approximation to the worn or artificially aged varnish regions of violins, we examined the influence of the applied pretreatments independently of the final varnish systems. The results are discussed in light of changes in vibrational properties as studied by modal analysis showing the coating system induced changes in eigenfrequency and damping, which is of high relevance for luthiers, who commonly tune the violin plates before varnishing. The results reveal the relationship between the coating systems’ influence on the sorption and vibrational behavior, which must be expected and taken into account for violins.

## Results

**Figure 2:**
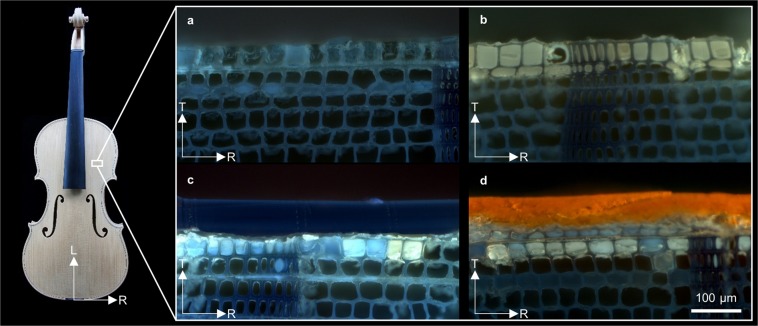
UV-light microscopy images of a wood samples treated with (**a**) sodium nitrite solution and a sealing (N + S) (**b**) nitrite solution and grounding (N + G) (**c**) nitrite solution, sealing, grounding and four layers of oil varnish (N + S + G + O) (**d**) nitrite solution, sealing, grounding and four layers of an alcohol varnish (N + S + G + A). The images were obtained on the same test samples that were used for neutron imaging (not from a violin) and do not show a subsequent treatment of one sample but samples after individual treatments. (L: longitudinal, R: radial and T: tangential wood direction).

For a systematic study on the influence of different varnish materials and their build-up on the sorption behavior, we investigated several wood penetrating treatments (Vernice Bianca (B), sodium nitrite solution (N), N+ sealing (S), N + grounding (G)), their combination (N + S + G) and entire coating systems with additional varnish layers on top (N + S + G + alcohol varnish (A), N + S + G+ oil varnish (O)). UV-light microscopy revealed the interface of the coatings and the wood surface (Fig. 2). Sealing and grounding penetrated into the wood structure and commonly occluded cell lumina in the first 1–2 cell rows. The additional oil and alcohol varnishes formed a homogenous covering layer on top (around 40–70 µm thickness). In contrast to the indications of the local images of sealing (Fig. 2a) and grounding (Fig. 2b), the sealing resulted in an on average, but statistically not significant, higher mass increase (cf. Table 1).

**Table 1:** Ratios of MC changes and induced mass changes Δm (and their standard deviation) for the different coating systems.

Treatment	B	N	N + S	N + G	N + S + G	N + S + G + A	N + S + G + O
$\frac{\overline{\Delta MC}}{\overline{{\Delta {\rm{MC}}}_{reference}}}$ (-)	1.00 (±0.02)	1.01 (±0.03)	0.72 (±0.07)	0.87 (±0.03)	0.65 (±0.03)	0.51 (±0.07)	0.52 (±0.06)
Δ*m* (mg)	7.8 (±6)	−1.6 (±1.1)	19 (±5)	11 (±7)	33 (±5)	69 (±5)	70 (±3)

The MC ratios were calculated with a least-square fitting for the entire MC changes (for A_tot_) during the 5 h sorption phase for the five different measurements. Based on a one-way analysis of variance with 5% significance value, Vernice Bianca and sodium nitrite solution, in contrast to all other coating systems, showed no significant influence. Further, comparing all applied coatings to each other, only the changes induced by N + S and N + S + G as well as the induced changes of the complete coating systems (N + S + G + A and N + S + G + O) were not significantly different. With regard to the mass changes, only the sodium nitrite solution did not result in a significant influence compared to the changes occurring for the reference samples (Δm_ref_ = −1.2 ± 1.1 mg). Further, the measured changes for the grounding in comparison to sealing and Vernice Bianca, as well as the changes for the complete coating systems were not significantly different. (B: Vernice Bianca, N: sodium nitrite solution, S: sealing, G: grounding, A: alcohol varnish and O: oil varnish).

### Coating systems as moisture barriers

For studying the sorption and desorption behavior by neutron imaging each measurement consisted of a humidity cycle, starting from 35% RH to 5 h at 95% RH and back to another 5 h at 35% RH. The local change in moisture content was calculated by a comparison of the neutron transmission radiographs at different relative humidities.

**Figure 3:**
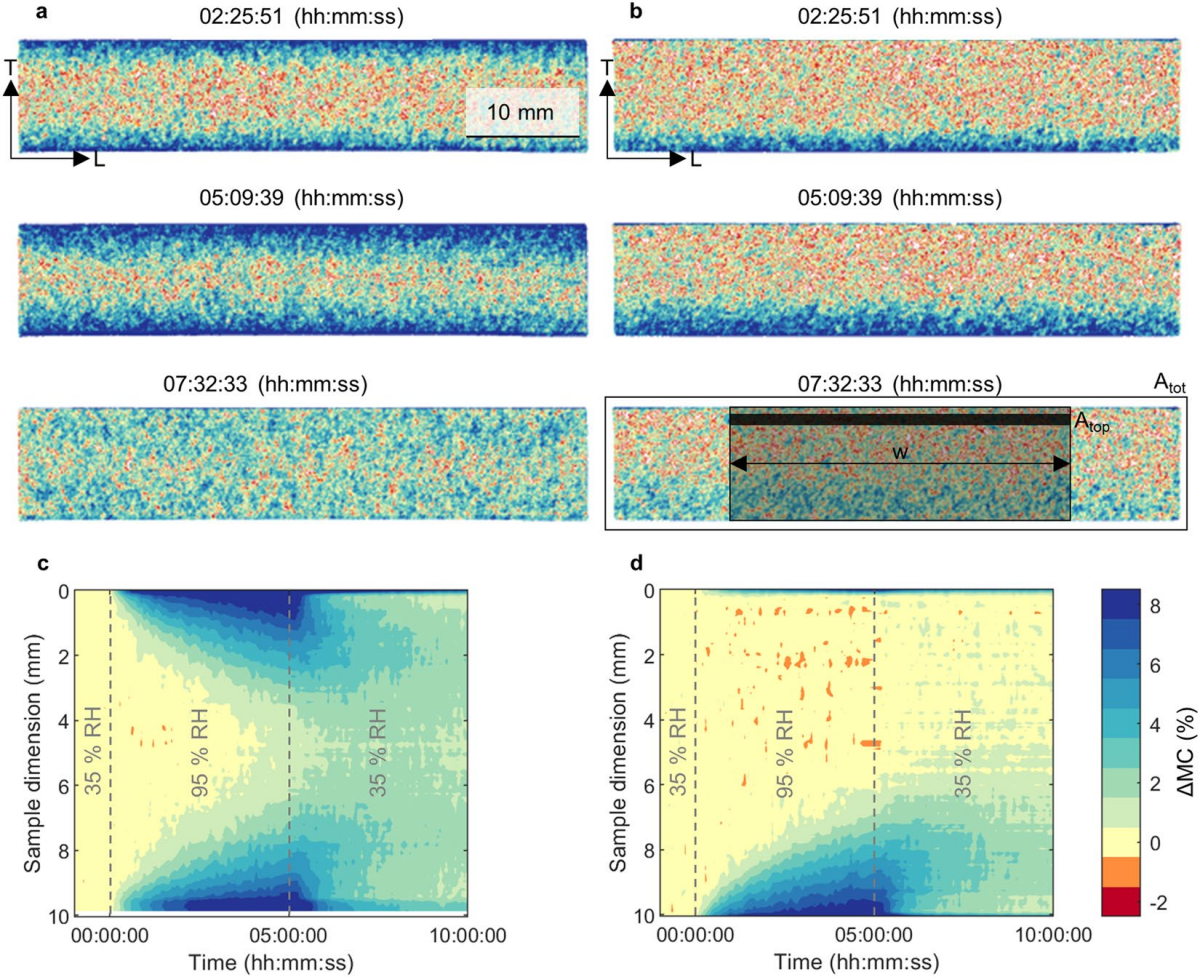
Spatial changes in MC compared to the initial equilibrated wood MC at 35% RH for (**a**) an unvarnished control and (**b**) a sample treated with a multi-layer varnish system with four layers of alcohol varnish on the top surface (N + S + G + A) after 2.5 h (top) and 5 h (middle) at 95% RH and after 2.5 h of desorption (bottom) (humidity was decreased to 35% RH after 5 h). Change in MC profiles from the top (0 mm) to bottom surface (10 mm), averaged over the width w = 30 mm for (**c**) the unvarnished reference sample and (**d**) the sample with complete varnish system. The averaging along w leads to a reduced noise for the results and allows a more detailed visualization of the changes over time. (L: longitudinal and T: tangential wood direction).

The recorded 2D spatial MC distributions clearly revealed the moisture barrier effect of the coating and the general sorption dynamics, when exemplarily comparing an unvarnished sample (Fig. 3a) and a wood sample only varnished on the top (N + S + G + A, Fig. 3b). For the unvarnished control, sorption occurred at top and bottom, whereas the multi-layer coating at the top of the varnished sample prevented any noticeable MC changes. From the moisture content profiles over time it is apparent that the samples had not reached an equilibrium moisture content at the end of the sorption phase, after 5 h at 95% RH (Fig. 3c,d). Hence, during the desorption phase the moisture content in the center of the samples still increased, while in the outer region the moisture content was decreasing. The thin blue lines at the top and bottom surfaces in Fig. 3c,d are artifacts caused by dimensional changes of the samples (swelling and shrinking) resulting in small dislocations of the wood surfaces. The time-dependent evaluations (as in Fig. 3c,d) of all coating systems are shown in the Supplementary Information.

**Figure 4:**
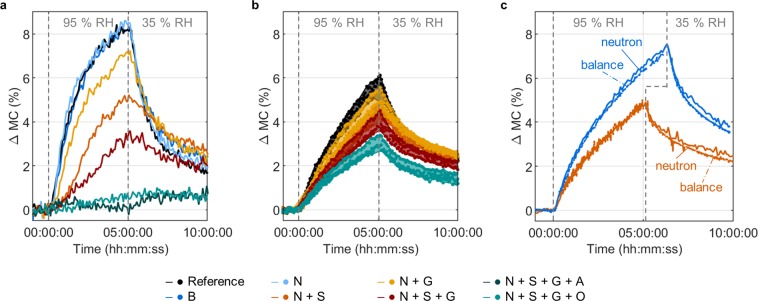
Changes in MC for the different applied varnish systems, spatially averaged over (**a**) an area, located at a distance from 0.5 to 1.5 mm below the treated top surface (A_top_) and thus showing a local analysis for one measurement, and (**b**) A_tot_, the entire samples, for three measurements. The variance between the different measurements (shaded regions in **b**) resulted from the variation in the natural wood properties, the applied coating systems and the actual conditions in the climatic chamber. (**c**) *In-situ* neutron and precision balance measurements of the entire MC changes (for A_tot_) of two individually measured samples evaluated over time. (B: Vernice Bianca, N: sodium nitrite solution, S: sealing, G: grounding, A: alcohol varnish and O: oil varnish).

A direct comparison of the time dependent effect of all different coating systems on the moisture content change was performed locally for the A_top_ region located directly below the treated top wood surface, and for the entire sample for the area A_tot_ (Fig. 4). The results of one measurement (Fig. 4a) exemplary display the general trend which was observed in the area below the top surface of the wood samples for the different coating systems. An individual treatment by Vernice Bianca (B) and sodium nitrite solution (N) did not result in a change of the sorption behavior when compared to control samples. Grounding resulted in reduced water uptake; however, it was less well protected than after sealing, whereas a combination led to improved protection. These differences, however, cannot directly be related to the different varnish materials, as, due to its higher viscosity, the final applied amount of particulated grounding was, albeit not significantly, lower than the applied amount of sealing (cf. Table 1). Nonetheless, the results show that the performance of the different pretreatments varies greatly, even for the limited number of samples investigated here. Moreover, only a fully varnished system with a top coating that is either alcohol or oil based resulted in marginal moisture content changes and a strong protective effect (Fig. 4a). Similar to the sorption phase, the sealing and grounding showed a retarding effect on moisture diffusion during the desorption phase. At the end of the measurements the moisture content was found to be higher when compared to the starting point, showing that the diffusion dynamics are faster during sorption than during desorption. The entire moisture content change analyses revealed the same trend as found in the local analyses below the treated surfaces when comparing a selection of different coating systems (Fig. 4b). However, the impact of the different coatings appeared to be less pronounced, which can be explained by the wood sample treatment in which only the top surface was coated and the bottom surface remained unvarnished. Nevertheless, the impact of sealing, grounding and complete coating systems are significant. Results show a 50% reduction in moisture uptake for the fully varnished wood samples when compared to the unvarnished control samples (Table 1).

An *in-situ* comparison of the entire moisture content changes, obtained by analyzing the neutron transmission data and the changes obtained with a precision balance for individual samples, showed good agreement and confirmed the measured values (Fig. 4c).

### Influence of coating build-up on vibrational properties

**Figure 5:**
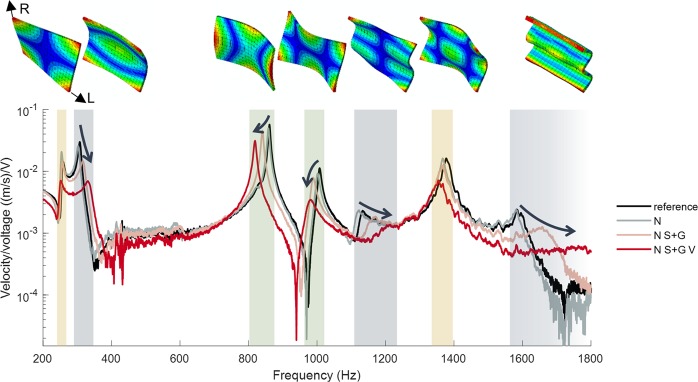
Frequency response functions (FRFs) of a spruce wood plate with dimensions of 140 × 100 × 2.9 mm^3^ (longitudinal × radial × tangential wood direction) during the varnishing process (control: unvarnished, N: after application of sodium nitrite solution, N S + G: after application of sealing and grounding, N S + G V: after application of four oil varnish layers). The radial bending modes are highlighted by the gray, the longitudinal bending modes by green and the torsional bending modes by yellow color, respectively. (L: longitudinal and R: radial wood direction).

Frequency response functions (FRFs) obtained by laser vibrometer modal analysis demonstrate the changes of the vibrational properties during a complete coating system application on a spruce plate (Fig. 5). As for violins, where the violin body functions as an amplifier that transmits the strings’ excitations into radiating sound, the FRFs describe the response of the plate to an excitation at different frequencies. The characteristics of this response, i.e. the location and width of peaks that indicate eigenfrequencies and their damping, depend on the geometry and the material properties (i.e. density, E-moduli and damping) of the structure and determine its final characteristic timbre.

The chosen geometry allows the investigation of different eigenmodes over a wide frequency range on flat plates as a first approximation to violin plates. The varnish layers were applied in the same manner as on the neutron samples, and the measurements were performed after the layers had been dried prior to the application of the next layer (after approximately 24 h). The individual eigenfrequencies are influenced in different ways due to the anisotropic mechanical properties of spruce, but the influences of subsequent varnishing steps did not counteract each other and thus they supplement the final impact of the complete system. The freshly applied coating system led to a pronounced increase in eigenfrequencies of the radial bending modes, a decrease in eigenfrequency of the longitudinal modes and to only minor changes in eigenfrequency for the torsional modes. The damping behavior is generally strongly increased by the varnish application. Also, the impact of the coating on the damping was most pronounced in radial bending modes. These general trends were evaluated in more detail for different kind of coatings^31^.

## Discussion

Using high spatial resolution and accuracy, this study has shown that the build-up of violin coatings has a strong impact on the sorption dynamics of the wood material. Incomplete coating systems result in significantly reduced protection against moisture uptake even during short range humidity changes. The latter finding is of crucial relevance to musicians when playing string-instruments in concert halls, where in addition to the direct moisture and sweat impacts from the player, rapid changes in relative humidity are encountered^32^. As a consequence, moisture uptake in the wood can influence wood properties, ranging from dimensional changes to even cracking, having a severe impact on the mechanical and vibrational behavior, which is even more pronounced in high wood moisture content gradients along the cross-section^33–35^. Hence, it appears to be favorable to apply thick and robust multi-layer varnish systems to obtain an effective barrier function. However, the application of multi-layer varnish systems is normally restricted to the outer surface of the instrument. The interior surfaces are commonly left unvarnished or treated with thin oil or protein containing layers. Our neutron imaging results indicate that a two-sided multi-layered application would likely result in superior protection, which would avoid cupping of the violin plates as observed for violins varnished on just one side^22^. Nonetheless, the moisture barrier effect is only one aspect of the influence of the coating for the violin. Our modal analyses have shown that a higher number of coating layers results in a stronger deviation from the original vibrational properties of the wood. This has considerable implications for luthiers who commonly tune the thickness of violin plates while in an uncoated state (‘in the white’), achieving unintended changes to sound quality at the final stage of product development. Although not directly measured, a two-sided coating system presumably has an even more pronounced effect on the vibrational properties detuning the delicate adjustment and diminishing the sound quality of the instrument. In conclusion, the varnish should be an effective moisture barrier with a minimum of layers and total coating thickness. While trading off the moisture protective effect of the coating against the varnish induced vibrational changes, our in-depth analysis confirms the traditional restriction of an application to the outer surface, which is more prone to moisture impact owing to its direct contact with the human body and sweat of the soloist^24^. Because only a complete coating system guarantees strong moisture protection, the wear and degradation of the varnish that occurs over time should be carefully observed. Associated changes in the dimensional response to varying humidity conditions, including the emergence of high local internal stresses in insufficiently protected regions, may become harmful to the instrument. In this case, required and appropriate protective measures, i.e. a professional re-varnishing of the instrument, could be advantageous.

With the possibility to accurately determine the moisture content distribution with high spatial and time resolution, the applied method shows high potential for further studies of interest in relation to wood sorption properties. Based on a more extensive study of additional varnish materials, specific statements on the relationship between the chemical and physical properties of varnish materials and their impact on the sorption process might be drawn. Possible further applications range from investigations of top and back plates of violins to an evaluation of wood treatments to lower the hygroscopicity of (tone)wood, as described in e.g.^21,36–38^.

## Methods

### Varnishing procedure

40 tone-wood spruce samples (5 times repetition of 8 different coating systems) with a mean density of 400 kgm^−3^ (±14 kgm^−3^, at 20 °C and 35% RH) and dimensions of 50 × 10 × 10 mm^3^ (L × R × T) were excised as twin samples. The lateral sides were sealed with an aluminum tape to ensure sorption at the varnished top and unvarnished bottom surfaces. By choosing a thickness of 10 mm, in contrast to typical thicknesses of soundboards in the range of 2.5 mm to 3 mm, and thus decoupling the sorption process at the top and bottom surfaces, the specific impact of the varnish systems on moisture diffusion could be investigated and illustrated. Referring to historical vanish systems and terminology^1^, different varnish materials and build-ups were studied: (i) B: Vernice Bianca, based on gum arabic, water and egg white, (ii) N: sodium nitrite solution with 4.3 wt% NaNO_2_ dissolved in distilled water functioning as a tanning stain, (iii) N + S: a clear oil varnish, consisting of mastix resin solved in turpentine labelled as sealer, (iv) N + G: a clear oil varnish, consisting of amber solved in linseed and polymerized linseed oil in combination with 29 wt% pumice powder termed as grounding, (v) N + S + G, (vi) N + S + G + A: an alcohol varnish based on shellac, gum elemi and spike oil dissolved in ethanol and known as 1704 varnish and (vii) N + S + G + O: an oil varnish, based on copals solved in turpentine and linseed oil. The pretreatments (B, N, S and G) were applied as single layers, while for the main varnishes (A and O) four layers were applied with a brush. Each fresh layer was exposed to 8 h UV light and subsequent layers were brushed in 24 h distances. The last layer was applied two weeks prior to the neutron measurements to ensure a complete hardening of the varnish systems. The choice of varnish materials and its processing followed the approach and suggestions of Ulrike Dederer, an experienced luthier, and are considered as representative traditional varnish materials (cf.^3,39,40^). Other frequently applied varnish materials could likewise been investigated but were not considered in this study. While keeping a limit to the number of different coatings, the studied combinations comprise different compositions that are widely applied but also subjected to some debate (e.g. used solvent, application of particulate ground). The varnish induced mass changes were measured with a precision balance (±1 mg) at 35% RH and 20 °C by weighting the sample before and after the varnish system application.

### Neutron imaging measurements and data post-processing

The measurements were conducted at the thermal neutron imaging beamline NEUTRA at PSI with an collimator ratio of L/D = 350^41^, a sample to detector distance of l = 30 mm and a resulting geometric unsharpness of u_g_ = l/(L/D) = 85.7 µm. To ensure equilibrated and known reference conditions, the samples were preconditioned at 20 °C and 35% RH. Each measurement consisted of eight samples, placed on an aluminum sample holder (Fig. 1a,c) and positioned in a climatic chamber for air-conditioning with a moisture generator and thermoelectric modules^42^. After measuring the reference conditions (35% RH) for 1 h, the humidity was increased to 95% RH for 5 h (sorption phase) and then returned to 35% RH for an additional 5 h (desorption phase). One radiograph with an exposure time of 15 s was taken every 5 min, resulting in time-resolved measurements of the spatial MC changes. The images were acquired with a 50 µm thick Li/F scintillator and an Andor NEO sCMOS camera (2560 × 2160 pixels, pixel size: 61.3 µm). For the weight changes’ validation measurements, single samples were placed on a sample holder on a precision balance (±0.1 mg accuracy, pixel size 48.5 µm) (Fig. 1b). During the 5 h sorption phase (at 95% RH), the balance measurements were corrected by adding 0.0359 g. This artefact is assumed to result from changing buoyancy forces acting on the sample holder due to changing air conditions and could be reproduced on the empty sample holder. Open beam, dark current and black body^43,44^ corrections were applied for image normalization in order to account for beam and detector inhomogeneities, background noise of the camera and scattered neutrons, respectively. Out of the resulting transmission images, the samples were separated, segmented, horizontally aligned and translated to the image center to enable a direct pixel-wise comparison of the changes over time. Generally, the MC describes the moisture induced relative mass change of wood compared to the oven-dry state:

1 $${\rm{MC}}=\frac{{\rm{m}}-{{\rm{m}}}_{{\rm{oven}}{\rm{dry}}}}{{{\rm{m}}}_{{\rm{oven}}{\rm{dry}}}}$$

With the water density r_h_ = 1000 kgm^−3^, the water neutron attenuation coefficient Σ_h_ = 3.6 cm^−1^ ^44^ and knowing the oven-dry wood density r_w_ and thickness d_w_ of the samples, the spatial absolute change in wood MC compared to the reference conditions (with an equilibrium MC of 9%) can be calculated by a comparison of a transmission image T to the transmission image of the reference conditions T_ref_^45^:

2 $$\Delta MC=MC-M{C}_{ref}=-\frac{{\rho }_{h}\,\mathrm{ln}(\frac{T}{{T}_{ref}})}{{\rho }_{w}{d}_{w}{\Sigma }_{h}}$$

### Modal analysis with laser vibrometer

The frequency response functions (FRF) were measured on a free rectangular spruce plate, suspended at the first vibration nodes on nylon cords, with dimensions of 140 × 100 × 2.9 mm^3^ (L × R × T). The vibration was excited contact-free by a loudspeaker positioned behind the plate. A PSV-500 scanning vibrometer was used to measure the vibration velocity.

## Supplementary information

Supplementary information
