# Supplementary material for: The influence of multi-layered varnishes on moisture protection and vibrational properties of violin wood
Source: Sci Rep. 2019 Dec 9;9:18611. doi: 10.1038/s41598-019-54991-5 (PMC6901594; doi:10.1038/s41598-019-54991-5)
Supplement: Supplementary file 1 — Supplementary information [file 41598_2019_54991_MOESM1_ESM.pdf]

Supplementary information to:

The influence of multi-layered varnishes on moisture protection and vibrational properties of violin wood

Sarah L. Lämmlein [1,2], David Mannes [3,*], Bart van Damme [1], Francis W.M.R. Schwarze [4], Ingo Burgert [1,2]

[1] Swiss Federal Laboratories for Materials Science and Technology (Empa), Dübendorf, Switzerland

[2] Swiss Federal Institute of Technology Zürich (ETH Zürich), Zurich, Switzerland

[3] Paul Scherrer Institute (PSI), Villigen, Switzerland

[4] Swiss Federal Laboratories for Materials Science and Technology (Empa), St. Gallen, Switzerland

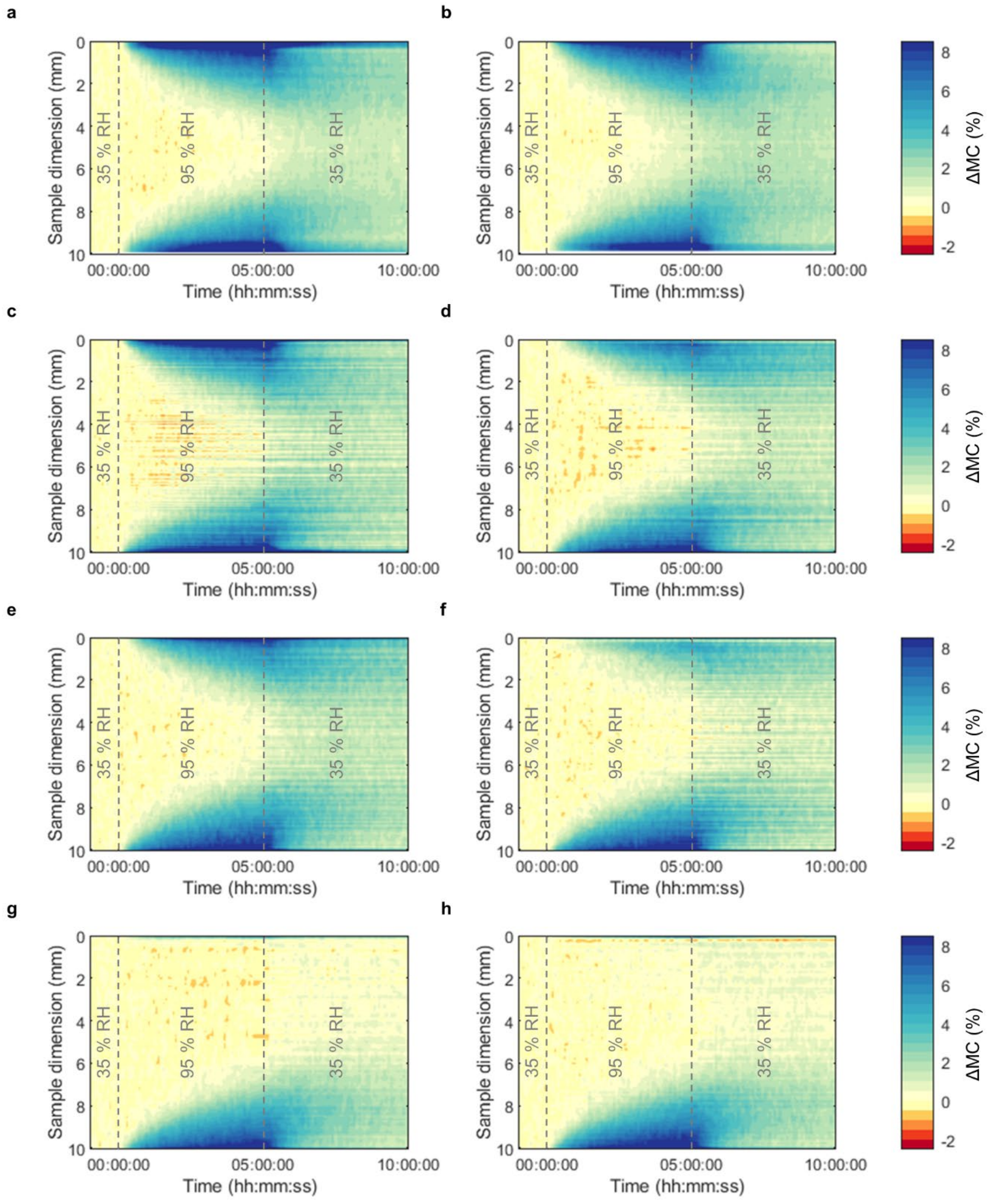

Supplementary figure 1 First measurement series. Changes in MC profiles from the top (0 mm) to bottom surface (10 mm), averaged over the width w=30 mm (cf. Fig. 3) for samples treated with **a** Vernice Bianca (B), **b** an unvarnished control, **c** sodium nitrite solution (N), **d** N and sealing (S), **e** N and grounding (G), **f** N+S+G, **g** N+S+A and four layers of alcohol varnish (A) and **h** N+S+G and four layers of oil varnish (O).

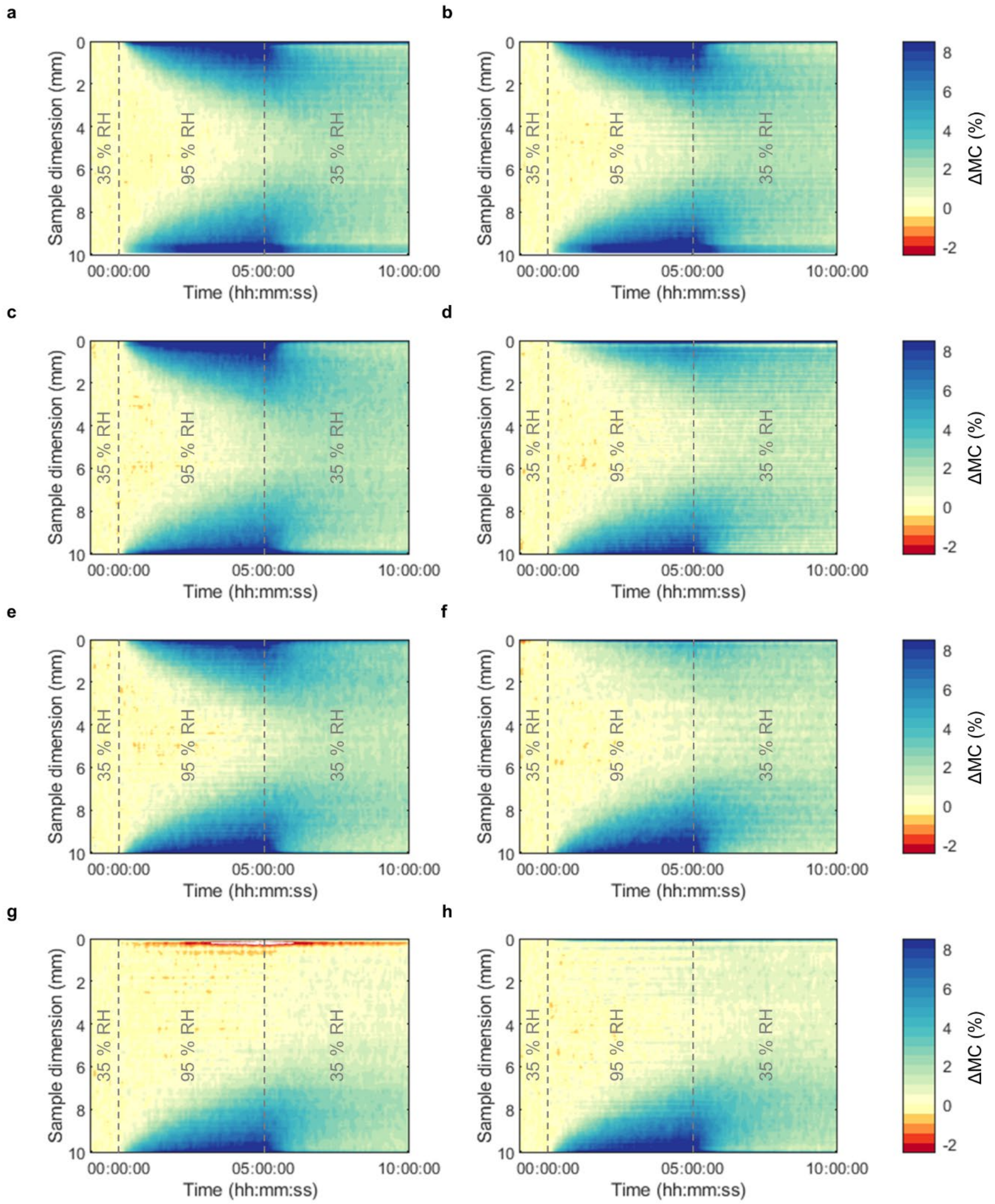

Supplementary figure 2 Second measurement series. Changes in MC profiles from the top (0 mm) to bottom surface (10 mm), averaged over the width w=30 mm (cf. Fig. 3) for samples treated with **a** Vernice Bianca (B), **b** an unvarnished control, **c** sodium nitrite solution (N), **d** N and sealing (S), **e** N and grounding (G), **f** N+S+G, **g** N+S+A and four layers of alcohol varnish (A) and **h** N+S+G and four layers of oil varnish (O).

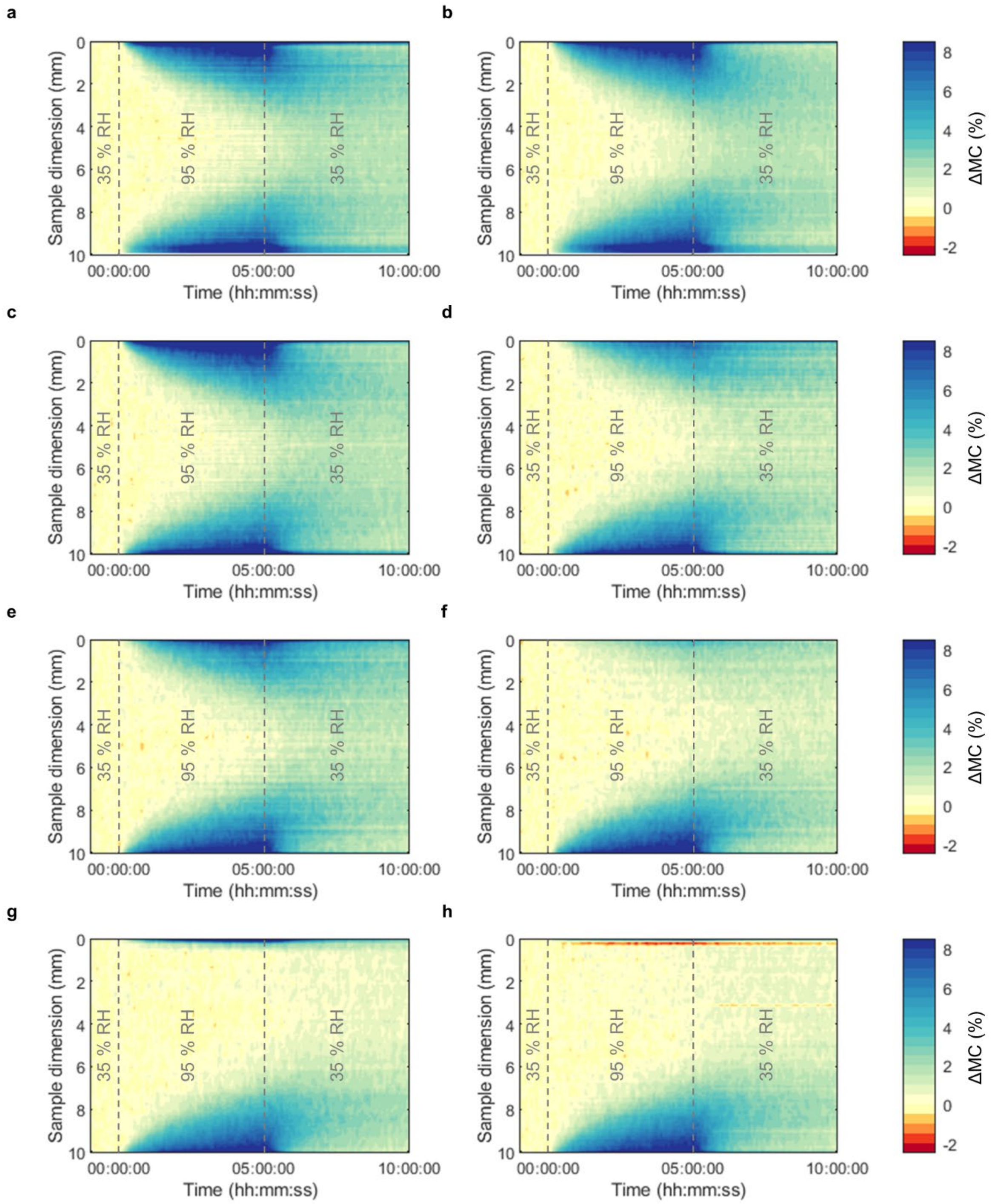

Supplementary figure 3 Third measurement series. Changes in MC profiles from the top (0 mm) to bottom surface (10 mm), averaged over the width w=30 mm (cf. Fig. 3) for samples treated with **a** Vernice Bianca (B), **b** an unvarnished control, **c** sodium nitrite solution (N), **d** N and sealing (S), **e** N and grounding (G), **f** N+S+G, **g** N+S+A and four layers of alcohol varnish (A) and **h** N+S+G and four layers of oil varnish (O).

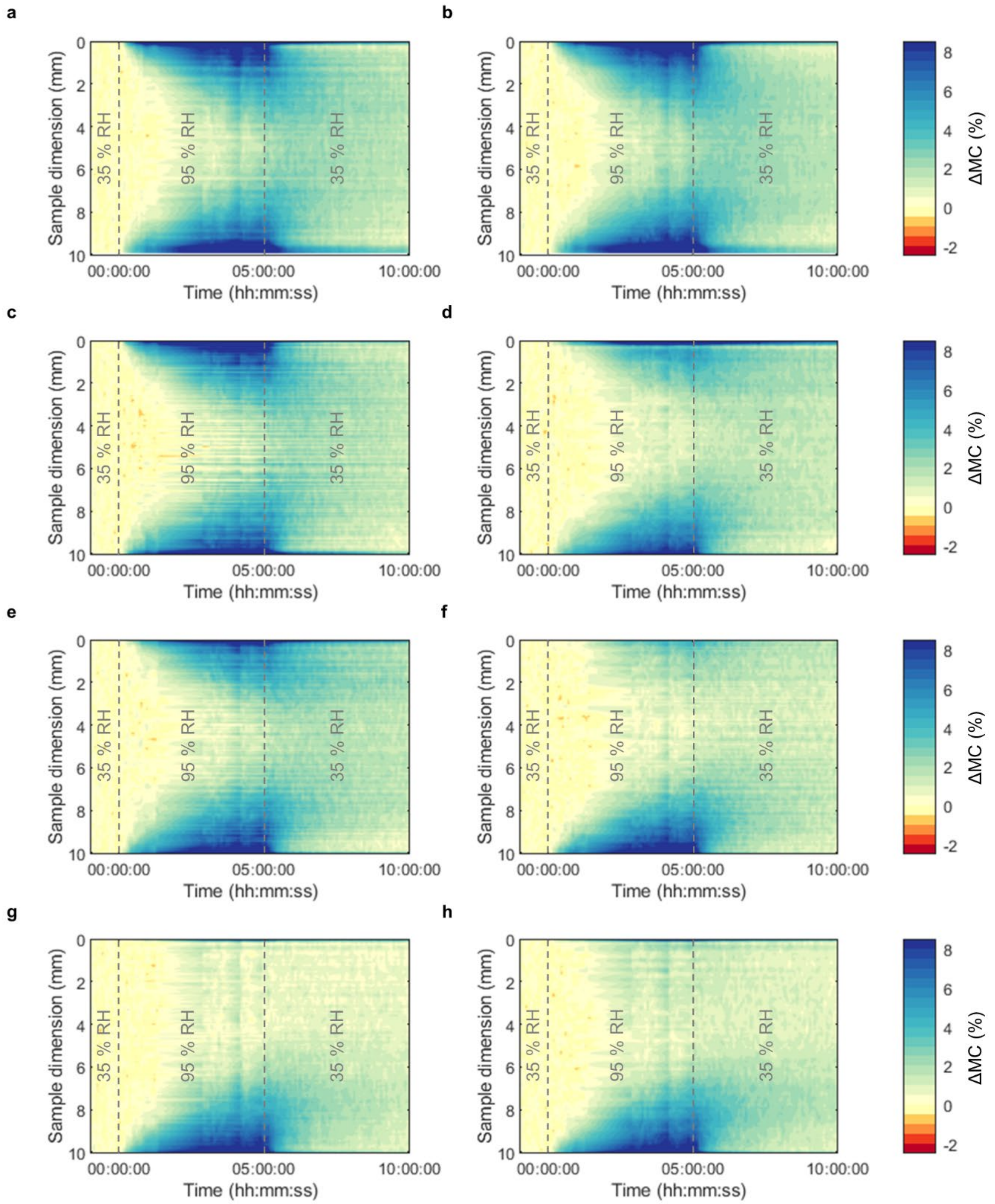

Supplementary figure 4 Fourth measurement series. Changes in MC profiles from the top (0 mm) to bottom surface (10 mm), averaged over the width w=30 mm (cf. Fig. 3) for samples treated with **a** Vernice Bianca (B), **b** an unvarnished control, **c** sodium nitrite solution (N), **d** N and sealing (S), **e** N and grounding (G), **f** N+S+G, **g** N+S+A and four layers of alcohol varnish (A) and **h** N+S+G and four layers of oil varnish (O).

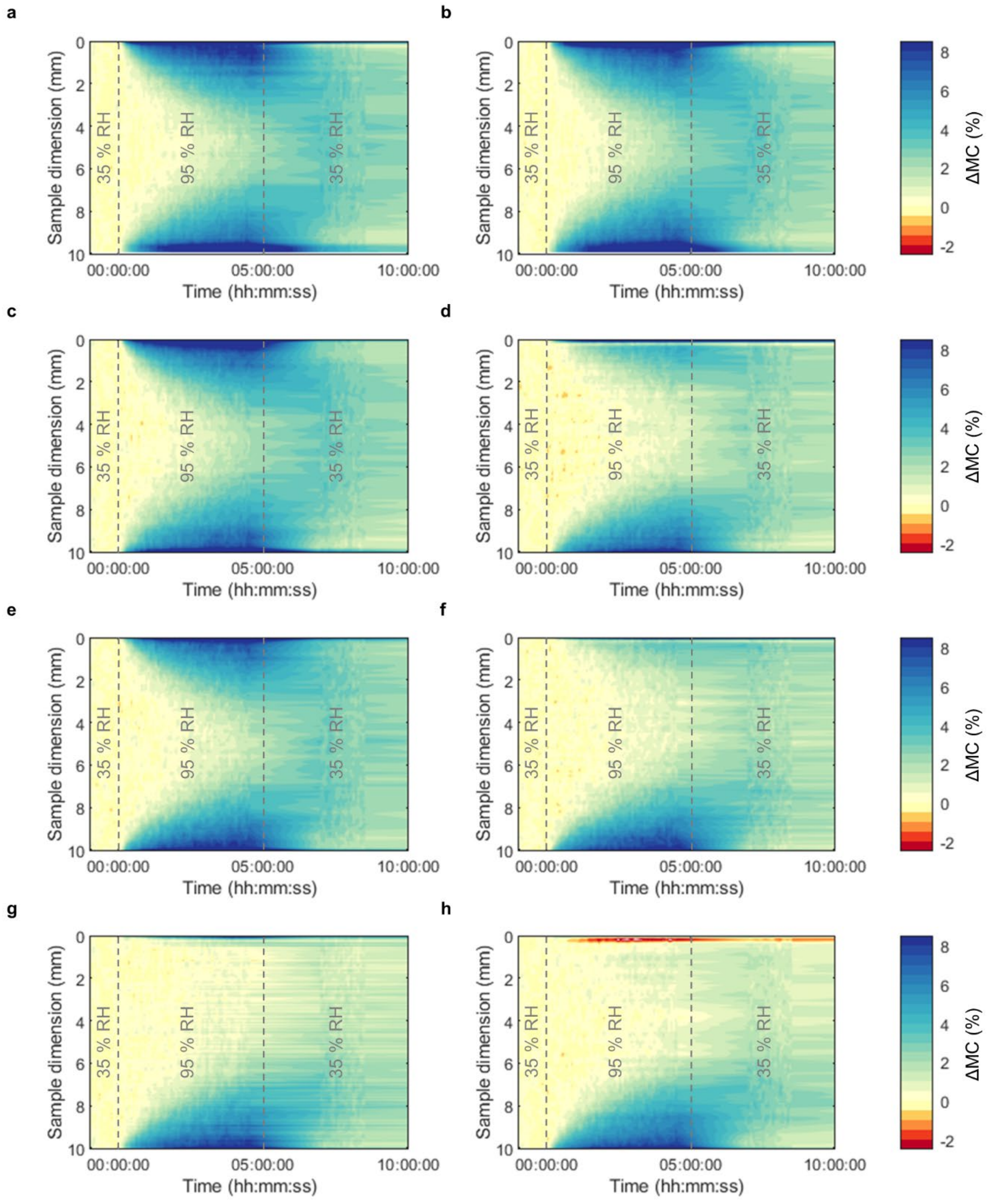

Supplementary figure 5 Fifth measurement series. Changes in MC profiles from the top (0 mm) to bottom surface (10 mm), averaged over the width w=30 mm (cf. Fig. 3) for samples treated with **a** Vernice Bianca (B), **b** an unvarnished control, **c** sodium nitrite solution (N), **d** N and sealing (S), **e** N and grounding (G), **f** N+S+G, **g** N+S+A and four layers of alcohol varnish (A) and **h** N+S+G and four layers of oil varnish (O).
